# Adsorption of RNA on mineral surfaces and mineral precipitates

**DOI:** 10.3762/bjoc.13.42

**Published:** 2017-03-01

**Authors:** Elisa Biondi, Yoshihiro Furukawa, Jun Kawai, Steven A Benner

**Affiliations:** 1 Foundation for Applied Molecular Evolution, 13709 Progress Boulevard, Alachua, FL, 32615, USA; 2 Firebird Biomolecular Sciences LLC, 13709 Progress Boulevard, Alachua, FL, 32615, USA; 3 Department of Earth Science, Tohoku University, 2 Chome-1-1 Katahira, Aoba Ward, Sendai, Miyagi Prefecture 980-8577, Japan; 4 Department of Material Science and Engineering, Yokohama National University, 79-5 Tokiwadai, Hodogaya-ku, Yokohama 240-8501, Japan; 5 The Westheimer Institute for Science and Technology, 13709 Progress Boulevard, Alachua, FL, 32615, USA

**Keywords:** carbonates, natural minerals, origins of life, RNA adsorption, synthetic minerals

## Abstract

The prebiotic significance of laboratory experiments that study the interactions between oligomeric RNA and mineral species is difficult to know. Natural exemplars of specific minerals can differ widely depending on their provenance. While laboratory-generated samples of synthetic minerals can have controlled compositions, they are often viewed as "unnatural". Here, we show how trends in the interaction of RNA with natural mineral specimens, synthetic mineral specimens, and co-precipitated pairs of synthetic minerals, can make a persuasive case that the observed interactions reflect the composition of the minerals themselves, rather than their being simply examples of large molecules associating nonspecifically with large surfaces. Using this approach, we have discovered Periodic Table trends in the binding of oligomeric RNA to alkaline earth carbonate minerals and alkaline earth sulfate minerals, where those trends are the same when measured in natural and synthetic minerals. They are also validated by comparison of co-precipitated synthetic minerals. We also show differential binding of RNA to polymorphic forms of calcium carbonate, and the stabilization of bound RNA on aragonite. These have relevance to the prebiotic stabilization of RNA, where such carbonate minerals are expected to have been abundant, as they appear to be today on Mars.

## Introduction

It has been nearly 70 years since Bernal broadly conjectured on possible roles of rocks and minerals in the assembly of complex organic species relevant to the origin of life [1]. This theme has now been revisited multiple times [2–3]. Rocks and minerals have been proposed to have had multiple roles that might have been productive for the emergence of Darwinism on Earth. Classically, these roles have included:

(i) Concentration. Whether they are delivered by meteorite or created on Earth, prebiotic organic molecules are expected to be dilute and, if concentrated, react unproductively with each other. Rock and mineral surfaces offer the opportunity to concentrate relevant species from dilute aqueous environments, perhaps without unproductive intermolecular reactions. Such adsorption as a concentration mechanism offers an alternative to evaporation in a desert environment. Mineral adsorption from a large ocean is an especially attractive alternative to desert evaporation for those who think that dry land was sparse on the early Earth [4].

(ii) Productive catalysis. Concentration is itself a way of "catalyzing" bimolecular reactions. However, rocks and minerals have also been considered as sources of conventional catalysis, where species on the surface of the mineral stabilize a transition state with respect to adsorbed ground state species [5].

More recently, and especially after the emergence of the "RNA first" hypothesis for the origin of Darwinism on Earth [6], rocks and minerals have been considered in other roles.

(iii) As inhibitors of reactions. One key problem obstructing the assembly of prebiotically productive organic species is the well-known propensity of organic molecules, especially those containing carbonyl groups such as carbohydrates, to react further to yield unproductive "tars". Mineral species, especially if they are slightly soluble into an aqueous environment, have been proposed to prevent classes of unproductive reactions [7–8].

(iv) Stabilizers. Many useful pre-biological polymers are subject to destruction by environmental forces, such as ultraviolet radiation and radioactivity. Adsorption of these onto mineral surfaces has been shown to slow that destruction [9], in some cases without greatly damaging the catalytic activity of those pre-biopolymers [10], in other cases with evolution [11].

As Hazen and Sverjensky remark [12], mineral environments are far more complex than the "Pyrex^®^ prebiotic chemistry" that dominates the field. However, in addition to creating an opportunity, this complexity creates problems, both intrinsic and experimental. For every constructive reaction that might be catalyzed by a mineral, the potential exists for that mineral to catalyze a destructive reaction. Further, although a mineral (by definition) is a pure substance, real minerals invariably have non-canonical elements incorporated within them; these defects may easily be the reason why a natural mineral adsorbs organic molecules or has an interesting reactivity. Further, even with an ideally pure mineral, the catalysis of interest can occur in defects in its crystalline surface. All of these problems are difficult to manage in a controlled laboratory environment.

How are we to explore this new complexity as we accommodate those who "plead" for a role for mineralogy in models for the origin of life? Two approaches are possible. On one hand, we might build a collection of natural minerals, and then run experiments on them with biopolymers having prebiotic interest, such as RNA. Unfortunately, natural minerals vary in chemical composition from specimen to specimen, and certainly from locale to locale. This is obvious even to an amateur. For example, natural calcium phosphate (apatite), of possible prebiotic interest as a source of the phosphate essential to prebiotic RNA synthesis [13], has different colors that reflect inclusion of different atomic species that are not in the canonical formula of the mineral.

Alternatively, reagents that have the components of those minerals, with exacting levels of purity, might be mixed in the appropriate ratio to create a synthetic mineral as a precipitate. Experiments might then be run on these synthetic minerals to study their interaction with biopolymers of interest, such as RNA. This approach has the advantage of offering exactly the kind of "controlled experiments" that chemists like. However, it is frequently criticized as being "artificial".

Even if this problem were to be mitigated or ignored, general chemical physics intervenes. Solid phases with high surface areas, and precipitates in particular, are general adsorbents, especially for macromolecules. Therefore, it is difficult to know, if RNA (for example) adsorbs onto a surface, whether the adsorption is in any sense specific, or whether it is just a general manifestation of big molecules adsorbing to big surfaces.

Here, we introduce a general strategy that mitigates some of these problems. The experiments measure the adsorbance of radiolabeled RNA onto binary inorganic species that have been obtained in two ways. In one, the species is precipitated as a synthetic mineral via a double decomposition reaction between the two mineral components. In the second, the mineral itself is obtained from a natural source, and the experiment measures the percentage of radiolabeled RNA bound to the natural mineral. In a third approach, two precipitated minerals are combined, and the partition of radiolabel RNA between the two is measured.

This strategy then asks whether the trend in radiolabeled RNA adsorption is consistent across their various forms and presentations, especially within a set of minerals having a common anion (for example, all carbonates) but differing in their cationic components (for example, magnesium carbonate, calcium carbonate, strontium carbonate, and barium carbonate). Here, we may even seek a Periodic Table trend, where adsorption changes consistently in a series of minerals as one of their elements is replaced by another element in a row or column of the Periodic Table.

Underlying these experiments is the following rationale: If the same trends are observed both in precipitated synthetic minerals as well as in natural minerals, and if radiolabeled molecules are partitioned consistently between two mineral species precipitated together, the effects cannot easily be nonspecific as general adsorption of big biopolymers onto big surfaces.

We report here the first cases where this rationale has been applied for RNA over a range of minerals. Surprisingly, some of these showed Periodic Table trends, in both their precipitated synthetic and natural forms. Further, we speculate that these trends can be accounted for by the changing size of the mineral lattice resulting from different ionic radii of different elements in a Periodic Table series.

## Results

### Carbonates

We examined first various binary carbonate minerals with Group II (alkaline earth) cations. These are interesting not only because carbon dioxide is likely to have been an abundant component of an early Earth atmosphere, but also because alkaline earths form a well-known set of binary carbonates that include insoluble magnesium, calcium, strontium, and barium carbonates (magnesite, calcite, strontianite, and witherite, respectively).

The magnesite specimen was from Minas Gerias, Brazil; the calcite was a specimen of "Iceland spar". The strontianite was obtained from the Minerva Mine in Illinois , and the witherite was obtained from Cave in Rock, Illinois. The specimens were washed with hydrogen peroxide (30%) followed by water and then ethanol to remove potential organic surface contaminants. The samples were then dried in air while covered.

To flat surfaces of the cleaned mineral were added droplets of an aqueous (unbuffered) solution of 5’-^32^P labeled 83-mer RNA (2 µL, 50 nM). This length was chosen because it is representative of lengths that Holliger, Joyce and others suggest is needed to initiate Darwinism [14–15]. Although shorter lengths have been recently shown to be able to assemble in longer molecules with replicase activity [16], these were not tested in this study. Data from Ferris’ lab suggest that, for adsorption on montmorillonite clays, longer RNAs adsorb better than short RNAs [17]. This deserves to be addressed systematically in a separate study.

After adsorption, the mineral surface was washed several times with H_2_O to remove unbound RNA. Then, the amount of RNA bound was calculated by subtraction of counts per minute in the washes.

**Figure 1:**
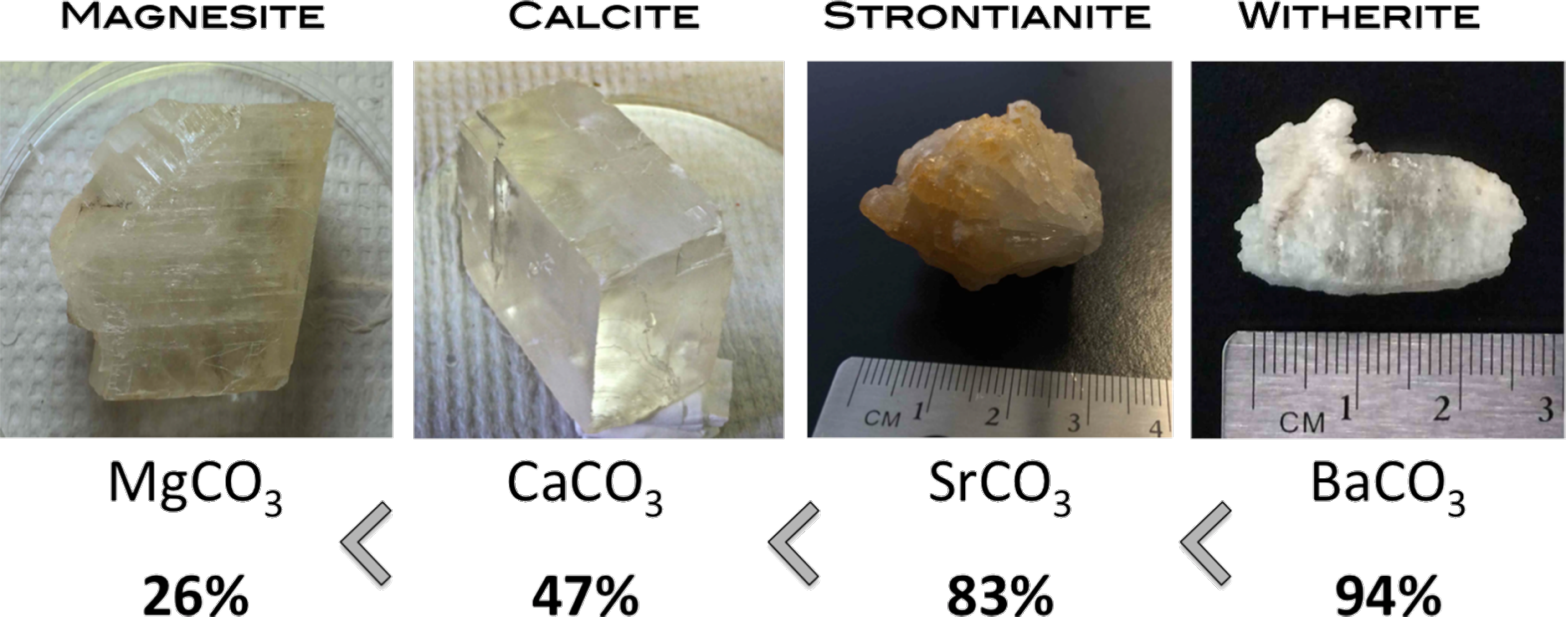
Adsorption of RNA on natural carbonate mineral samples.

The results are shown in Figure 1. Here, we were surprised to see a Periodic Table trend in these carbonates. Thus, while only a quarter of the radioactivity remained bound to the surface of the specimen of magnesite (with magnesium), ≈94% of the reactivity was bound to the surface of the specimen of witherite (with barium). The fraction bound to calcite (calcium) and strontianite (strontium) were intermediate, 47% and 83%. Thus, a Periodic Table trend is observed with the binding of RNA to the carbonates relatively Ba > Sr > Ca > Mg.

Following the dual-approach rationale, we then asked whether the same results could be qualitatively observed with precipitated synthetic minerals (Materials and Methods). These results are collected in Table 1. The same Periodic Table trend is observed with the precipitated synthetic carbonate minerals. Here, the percentage adsorption ranged from 95% to 77%, again with the ranking Ba > Sr > Ca > Mg. Again, the precise percentages have no easy interpretation (but see below). However, the fact that the same trend is observed with the precipitated synthetic minerals suggests that the trend with the natural minerals is not due to impurities in the natural species.

**Table 1:** Adsorption of RNA on synthetic minerals formed by double-decomposition reactions.^a^

	MgCl_2_	CaCl_2_	SrCl_2_	BaCl_2_	MnCl_2_	

Na_2_B_4_O_7_	no PPT	86%	87%	95%	87%	
Na_2_CO_3_	magnesite 77%	calcite 86%	strontianite 90%	witherite 95%	rhodochrosite 89%	
Na_2_PO_4_^b^	64%	apatite 93%	strontium apatite 84%	barium apatite 32%	metaswitzerite 86%	
Na_2_SO_4_	no PPT	gypsum 2%	celestine 71%	baryte 88%	no PPT	
Na_3_VO_4_	magnesium coulsonite 78%	cavoite 92%	73%	85%	ansermetite 38%	
Na_2_HAsO_4_	6%	johnbaumite 73%	4%	gurimite 30%	61%	
NaF	no PPT	fluorite no PPT	72%	15%	no PPT	

	FeCl_2_	FeCl_3_	CoCl_2_	NiCl_2_	CuCl_2_	ZnCl_2_

Na_2_B_4_O_7_	88%	no PPT	87%	94%	96%	93%
Na_2_CO_3_	siderite 65%	no PPT	cobalite 95%	94%	malachite 73%	smithsonite 93%
Na_2_PO_4_^b^	vivianite 68%	30%	pakhomovskyte 80%	75%	libethenite 84%	hopeite 6%
Na_2_SO_4_	22%	no PPT	no PPT	no PPT	no PPT	no PPT
Na_3_VO_4_	fervanite 2%	46%	2%	12%	73%	17%
Na_2_HAsO_4_	75%	10%	erythrite 12%	49%	lammerite 79%	adamite 62%
NaF	24%	29%	no PPT	no PPT	43%	no PPT

^a^No PPT: no precipitate observed. For some minerals, the name of the natural species is reported. ^b^Na_3_PO_4_ + NaHPO_4_.

**Figure 2:**
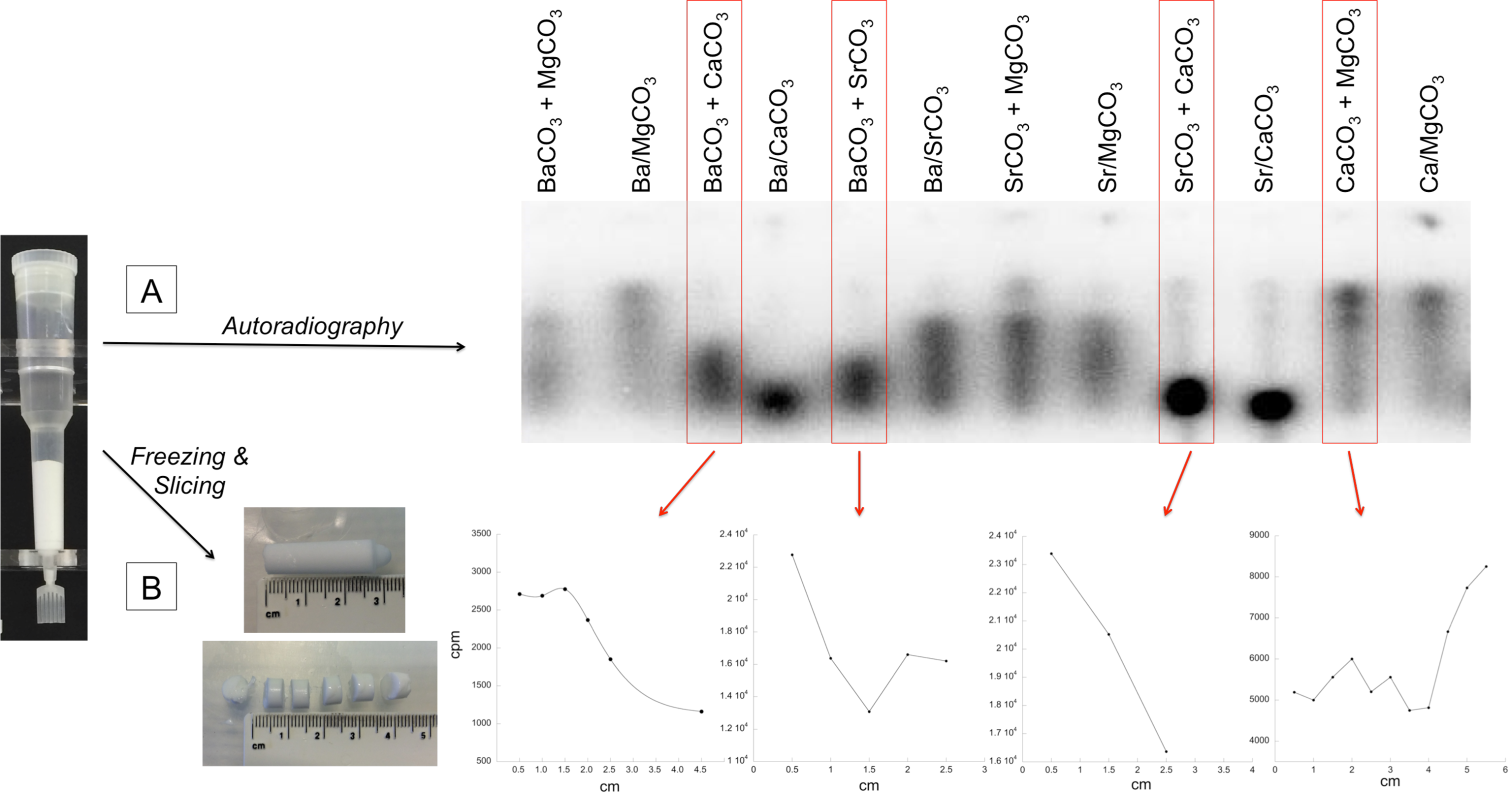
Co-precipitation experiments on carbonate minerals for RNA-binding competition. The precipitated column formed by two carbonates and containing radioactive RNA (left, see text for details) is exposed to a phosphorimager screen to observe RNA localization in the column (A). For each pair, carbonates are either prepared separately and then mixed together (“xCO_3_ + yCO_3_”, first column per pair), or all the ingredients are co-precipitated (“xyCO_3_”, second column per pair). Subsequently (B), columns xCO_3_ + yCO_3_ are frozen, cut in slices, and the radioactivity in each slide plotted versus the column height in cm. Columns for the pair Ba–Mg and Sr–Ca could not be frozen and sliced due to their high lability; their autoradiograms were profiled instead for RNA localization (data not shown). Carbonate density: CaCO_3_ 2.71 g/cm^3^ < MgCO_3_ 2.96 g/cm^3^ < SrCO_3_ 3.5 g/cm^3^ < BaCO_3_ 4.29 g/cm^3^.

To complete the analysis, we then co-precipitated various pairs of synthetic carbonates by mixing the appropriate aqueous solutions of the alkali metal chlorides with an aqueous solution of sodium carbonate in a 1:1 ratio (Figure 2). These were then easily separated gravitationally, as the different carbonates have different densities (CaCO_3_ 2.71 g/cm^3^; MgCO_3_ 2.96 g/cm^3^; SrCO_3_ 3.5 g/cm^3^; BaCO_3_ 4.29 g/cm^3^). The partition of RNA between each pair was then observed by pre-equilibration of the radiolabeled 83-mer RNA in a column with the two minerals, followed by dissection of the column and counting various slices within it. The labeled RNA partitioned as seen in the synthetic minerals precipitated individually: Ba > Sr > Ca > Mg.

### Sulfates

Binary alkaline earth sulfates are fewer in mineral form, since the first member of the Periodic Table series (magnesium sulfate, epsomite) is quite soluble in water. However, with precipitated synthetic minerals, the same trend was observed, with barium sulfate binding RNA more than strontium sulfate, which bound more RNA than calcium sulfate (Table 1). The corresponding trend was also observed in the specimens of the natural minerals, with baryte > celestine > gypsum (59% > 49% > 20%) (Table 2).

**Table 2:** Adsorption of RNA on all the natural minerals tested in this study.

Family	Mineral	Adsorption

carbonates	magnesite, MgCO_3_	26%
	calcite, CaCO_3_ aragonite, CaCO_3_	47% 76%
	strontianite, SrCO_3_	83%
	witherite, BaCO_3_	94%
	rhodochrosite, MnCO_3_	11%
	smithsonite, ZnCO_3_	5%
sulfates	gypsum, CaSO_4_	20%
	celestine, SrSO_4_	49%
	baryte, BaSO_4_	59%
phosphates & vanadates (apatite family)	apatite, Ca_2_(PO_4_)_3_Cl	28%
	vanadinite, Pb_5_(V/AsO_4_)_3_Cl	72%
	vivianite, Fe_3_(PO_4_)_2_	12%
arsenates	erythrite, Co_3_(AsO_4_)_2_	92%
	adamite, Zn_2_AsO_4_OH	2%
fluorites	purple fluorite, CaF_2_	no adsorption
	green fluorite, CaF_2_ + Fe or Sm inclusions	25%
borates	colemanite, CaB_3_O_4_(OH)_3_	31%
silicates	opal, SiO_2_	27%
	talc, Mg_3_Si_4_O_10_(OH)_2_	95%
	topaz, Al_2_SiO_4_(F,OH)_2_	33%
	amazonite, KAlSi_3_O_8_	31%
	mica, KAl_3_Si_3_O_10_(OH)_2_	22%
	beryl, Be_3_Al_2_Si_6_O_18_	17%
	olivine, (Mg,Fe)_2_SiO_4_	12%
	obsidian, SiO_2_+MgO+Fe_3_O_4_	8%
	danburite, CaB_2_(SiO_4_)_2_	no adsorption
	tourmaline, (Na,Ca)(Mg,Li,Al,Fe^2+^)_3_Al_6_(BO_3_)_3_Si_6_O_18_(OH)_4_	no adsorption
	agate, SiO_2_	no adsorption
	herkimer Diamond, SiO_2_	no adsorption
oxides	pyrite, FeS_2_	95%
	hematite, Fe_2_O_3_	30%
	rutile, TiO_2_	21%
	olivine, (Mg,Fe)_2_SiO_4_	12%
	magnetite, Fe_3_O_4_	no adsorption

### Constraints of natural mineralogy

However, and as a limitation of this approach, many minerals that might be made in the laboratory have no known natural correlates that we can examine in parallel. For example, in the synthetic borate minerals, the barium species also binds most tightly. However, to our knowledge, no natural strontium or barium borate mineral has been reported. The natural calcium borate mineral that we tested (colemanite) bound 31% of the RNA presented to it. Further, although the magnesium borate mineral is known naturally as boracite, we were unable to get a precipitate of the synthetic mineral by mixing magnesium chloride and sodium borate (Table 1).

#### Differential adsorbance need not proceed uniformly across the Periodic Table

While a Periodic Table trend is easy to observe, there is no reason a priori why such a trend should exist. For example, one might speculate that RNA would adsorb better onto a surface if the pattern of anion and cation sites on that surface matches more closely the distances of the anionic sites (phosphates) on the RNA molecule. While one might expect different cations in a mineral would change the spacing of those sites, there is no reason why the heaviest cation would have sites that match RNA the best. Indeed, if this were the mechanism for different surfaces having different affinities for RNA, one might expect within a Periodic Table trend to have a mineral that maximally absorbs somewhere in the middle of the series, rather the end of the series.

We may, in fact, see this in these data. For example, among the precipitated phosphates, the calcium species bound more RNA (93%) than the magnesium phosphate (64%), the strontium phosphate (84%), and barium phosphate (32%). While the calcium phosphate is well-known in the natural world in various forms (apatite), and while calcium is known to be replaced in natural minerals by strontium and barium to give species known as "strontium apatite" and "barium apatite", the strontium and barium forms are very seldom found in nature, and are not available for this kind of study.

The same comments apply to vanadates and arsenates, which we examined because of their structural resemblance to phosphates [18–19]. Here, the synthetic alkaline earth minerals showing the best binding are calcium vanadate and calcium arsenate. The synthetic transition element arsenates and vanadates that bind RNA best are both with copper. However, natural minerals that incorporate these specific atomic constituents are quite rare. For example, the most common vanadate mineral in museums (vanadinite) has lead as its cation. Vanadinite and calcium phosphate have analogous crystal forms (as do the lead arsenate mimetite and the lead phosphate pyromorphite). Further, vanadinite adsorbed RNA well (72%). However, lead strikes us as being an unlikely element to have been involved in prebiotic chemistry (but see refs. [20–22]).

#### Adding complexity

The alkaline earth carbonates and sulfates make conveniently simple systems where the natural-synthetic combination analysis can be easily applied. Other classes of minerals are more difficult to manage for two classes of reasons.

First, the cation(s) in the mineral may be redox active. Here, in a precipitation to give a synthetic mineral, the presence of oxygen can lead to a precipitate having the cation in mixed oxidation states.

Second, we cannot conveniently add a buffer to control the pH in an experiment that precipitates synthetic minerals; it would add an unnatural component into the system. This means that different anions with different protonation states (for example, H_2_PO_4_^−^, HPO_4_^2−^, and PO_4_^3−^) are, de facto, the buffering species in the precipitation experiments. Nevertheless, we collected data for a variety of natural species, including several that are not conveniently made by double decomposition reactions from water-dissolved salts. These are shown in Table 2.

For example, manganese carbonates (rhodochrosite) and zinc carbonate (smithsonite) were examined. Both adsorbed comparable amounts of RNA to their surfaces in the mineral specimens that were examined, 11% and 5% respectively. Comparable amounts of RNA adsorbed to each of the precipitated minerals (93% and 89%, respectively). However, it was difficult to find a rationale to compare these numbers across the Periodic Table to numbers obtained with the alkaline earth carbonates.

Finally, several silicates were examined for their ability to adsorb RNA. Silicates, of course, are represented by a very large number of minerals, and this work examined only a very small fraction of these. We recently reported work examining the adsorption and stabilization of RNA on opal [23].

#### Polymorphism

Another layer of complexity comes from the fact that the same set of atoms can form different crystal forms. For example, calcium carbonate can precipitate as calcite, aragonite, or vaterite. Calcite crystallizes in a trigonal space group; aragonite has an orthorhombic structure [24–25]. Calcite is the more stable and consequently most common phase, while aragonite is less stable and less common, although it does occur in nature as a metastable phase [26]. Vaterite, also μ-CaCO_3_, is a third metastable phase of CaCO_3_. It occurs much less commonly in nature because it is the least thermodynamically stable. It generally and rapidly transforms itself into one of the other two forms [27]. Vaterite is mostly seen when biological systems intervened to precipitate calcium carbonate. In forming the minerals synthetically, calcite dominates CaCO_3_ that precipitates upon mixing CaCl_2_ and Na_2_CO_3_ in water at near-neutral pH and room temperature and pressure; absent contaminants [28], aragonite is not formed. We easily reproduced this general result, establishing the structure of the precipitated phases that we obtained by both staining with Feigl’s stain (silver sulfate and manganese sulfate) [29] and by powder X-ray diffraction.

To complete our analysis of the CaCO_3_ system, we obtained natural specimens of the mineral aragonite and calcite. Experiments consistently showed that aragonite adsorbed more radiolabeled RNA than calcite. To obtain a synthetic mineral by precipitation, we reasoned that if RNA prefers to bind to aragonite over calcite, then perhaps RNA would nucleate the formation of an aragonite precipitate over a calcite precipitate.

**Figure 3:**
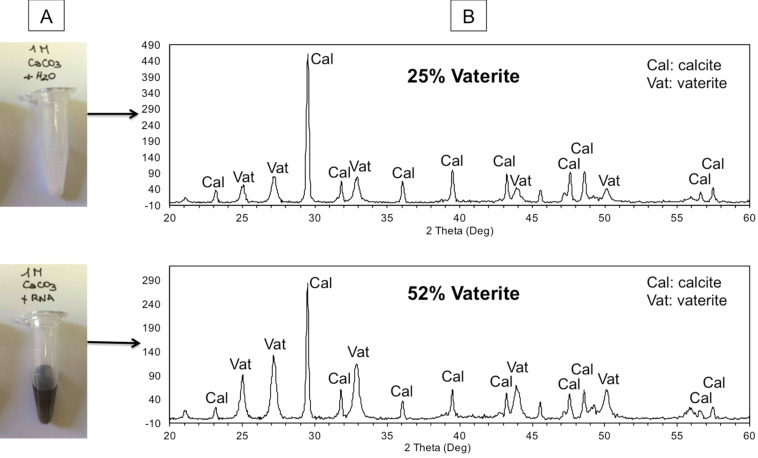
RNA-induced calcium carbonate polymorphism. A: Feigl’s stain of CaCO_3_ precipitate formed by double decomposition of 1 M CaCl_2_ + 1 M Na_2_CO_3_ in the absence (upper tube) or presence (lower tube) of RNA. B: X-ray powder diffraction of samples prepared the same way showing a net increase in vaterite versus calcite.

Initial results were auspicious. Feigl’s stain suggested that CaCO_3_ precipitated preferentially as aragonite in the presence of RNA, here isolated from *Aspergillus*. This was first observed when a solution of Na_2_CO_3_ (1 M) was mixed with a solution of CaCl_2_ (1 M) in the presence of 160 ng rRNA, with control experiments identical except for the absence of RNA. Both precipitates were stained with Feigl’s stain, with which aragonite is stained black, while calcite remains white (Figure 3). We then did powder X-ray diffraction to confirm the crystalline form of the precipitated calcium carbonate. Here, the results were variable, but the precipitate formed in the presence of RNA was often identified as being primarily vaterite. We do not have a molecular interpretation of these observations.

#### Stability of bound RNA

**Figure 4:**
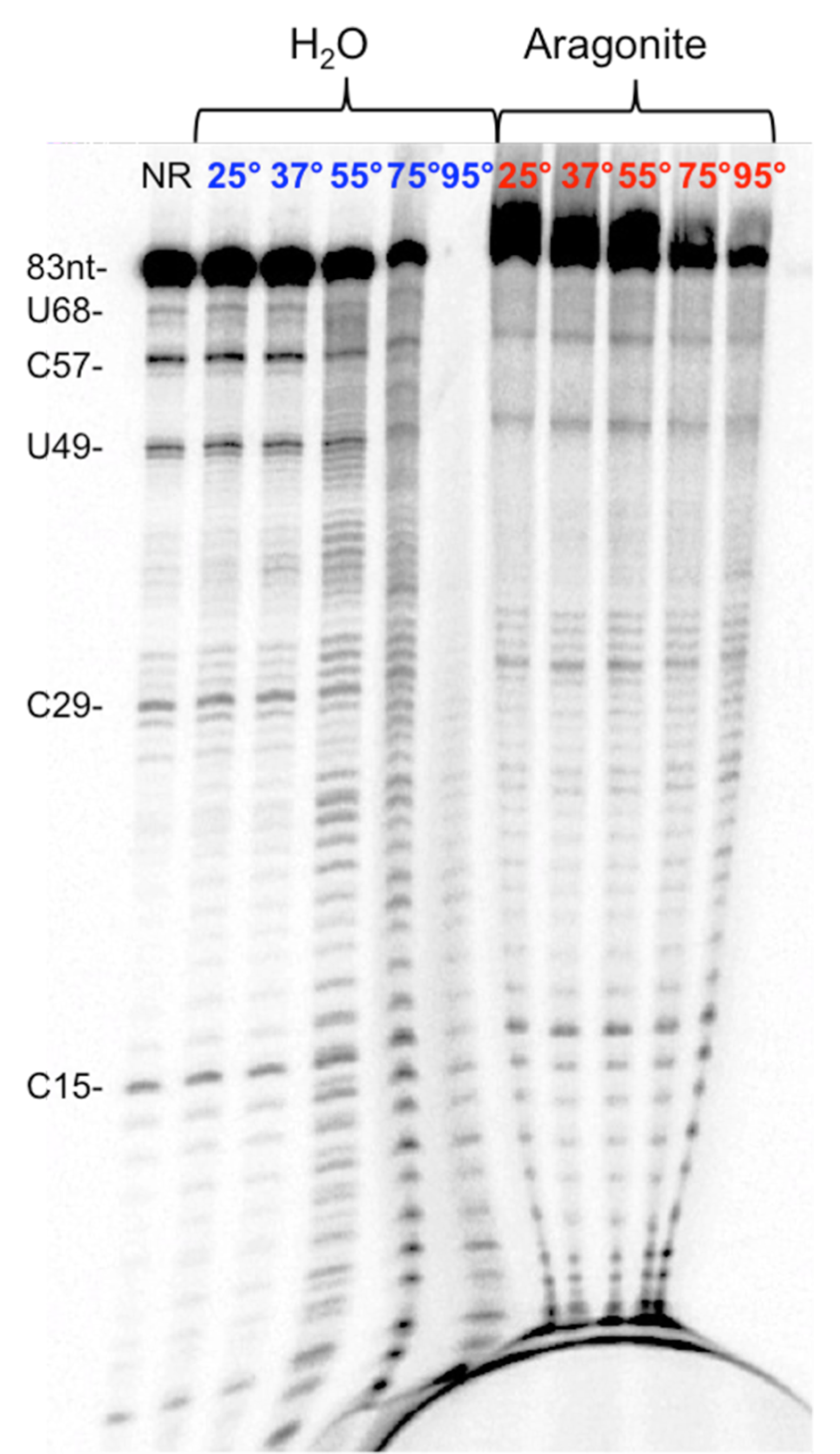
RNA adsorbed on aragonite is resistant to thermal degradation in aqueous solution. 18% denaturing PAGE of a 83-mer ssRNA incubated for 2 hours at 25, 37, 55, 75, and 95 °C, either free (left) or adsorbed to aragonite (right). Full length RNA and nucleotides in the sequence that are hot spots for degradation are indicated on the left.

We then showed that RNA bound to aragonite was more stable than the same RNA in aqueous solution. For these experiments, the same 5’-^32^P labeled 83-mer RNA (2 µL, 50 nM) was spotted on five pieces of natural aragonite, washed with H_2_O to eliminate unbound RNA, placed dry in a thermoblock at increasing temperatures (25 °C, 37 °C, 55 °C, 75 °C, or 95 °C), and incubated for two hours. After incubation, RNA was eluted from aragonite with 1 M formic acid, purified, and loaded on denaturing PAGE with a set of control samples where RNA was treated the same way, but in aqueous phase (see Materials and Methods). Interestingly, ≈70% of the RNA bound to aragonite remained full-length after incubation at 95 °C for 2 hours. In contrast, RNA treated the same way but in aqueous solution (Figure 4, compare lanes 6 and 11) showed high levels of degradation, with no detectable full-length RNA left.

## Discussion

The results reported here show that where it is possible, a comparison of the natural minerals, the synthetically precipitated minerals, and co-precipitated mineral combinations can be used to drive the conclusion that the adsorbance data collected are relevant to the mineral species themselves, and do not merely reflect the adherence of large macromolecules to large surfaces. This comparative approach also allows us to avoid a difficult discussion about what "percentage adsorbance" actually means in molecular terms, where the actual surface areas involved are essentially unknowable.

It should be noted that precipitated minerals are not necessarily (or even generally) amorphous materials. However, the size of their crystals is generally smaller than the size of crystals of minerals collected in the field.

Where possible (for example, multiple fluorite specimens, large homogeneous surfaces of calcite and magnesite, etc.), replicas were done. However, the main point presented here is that the error is not the kind of "error" that can be analyzed by standard statistical methods. This requires that the error be "normally distributed". Here, the error problems come from systematic errors relating to the natural samples, as two different exemplars of the "same" mineral, or even two different portions of the same specimen, may in fact be of different composition and thus may give different results. They are not Boltzmann "normally" distributed, and adding standard deviations from multiple runs provides only the deceptive illusion of statistical support. In this work, we circumvented this problem by asking whether the trend in radiolabeled RNA adsorption is consistent across the various forms and presentations of a mineral.

The most obvious limitation of this comparative approach comes from nature herself. The rarity of minerals having different elemental compositions determines their availability for these experiments. Some elemental compositions are simply not found in nature at all.

Thus, an analysis that involves Group (vertical) comparison of transition metal minerals in the Periodic Table is not particularly sensible using this approach. For example, iron phosphate is a well-known mineral (vivianite) and it adsorbs RNA (≈12%, Table 2). However, it does not make sense to seek the Periodic Table correlates of vivianite below iron. This would require us making and/or finding ruthenium phosphate and osmium phosphate, neither of which has been reported in mineralogy.

Likewise, horizontal comparisons across the Periodic Table are problematic, even within transition metals. For example, we found that RNA binds to natural titanium dioxide (rutile, 21% in our experiments) and iron(III) oxide (Fe_2_O_3_, hematite, 30% in our experiments), but not to magnetite (Fe_3_O_4_) [30]. However, the differences in redox states accessible to these different elements make direct comparison unlikely to be productive.

The most striking outcome of these results is the Periodic Table relationship in the adsorbance of RNA to alkaline earth minerals, both the carbonates and the sulfates. In both cases, the barium mineral bound more RNA than the strontium mineral, which bound more RNA than the calcium mineral, which bound more RNA than the magnesium mineral (when available).

Some evidence suggests that the crystalline surface is important in this trend. For example, like witherite and strontianite, aragonite adsorbs RNA better than calcite. Further, the crystal structures of witherite and strontianite belong in the same family as the crystal structure of aragonite. Likewise, vaterite resembles aragonite in its crystal structure more than either resembles calcite. Together, these results suggest, at least at the level of hypothesis, that the molecular structure of RNA is more compatible with the surface of an orthorhombic carbonate crystal (the “aragonite group”) having cations arranged with pseudo-hexagonal symmetry, than with the trigonal crystals observed in calcite and magnesite.

Here, the adsorbance of RNA on aragonite, and the ability of aragonite to nucleate the growth of aragonite and/or vaterite over the thermodynamically more stable calcite has potential prebiotic significance, as does the stabilization of RNA on these carbonates. All of these minerals are likely to have been present on early Earth. They are also known on Mars [31]. Today, most calcium carbonate is the result of biological activity. Where that activity is not present today (perhaps, but perhaps not, on Mars), we might expect to find stabilized RNA formed abiologically.

These results must remain tentative until reproduced by other laboratories, of course. We remain concerned that specific properties of natural minerals may differ with different sources, different impurities, different levels of success in cleaning their surfaces, and a thousand other variables that might influence these results [32]. Mitigating this concern is the fact that the patterns of adsorbance were unchanged in these experiments whether or not the mineral was cleaned by treating with hydrogen peroxide or diluted acid.

However, as a cautionary note, we point to the results (Table 2) that were obtained with different specimens of calcium fluoride (fluorite). Fluorites in nature are known for their dramatic and often attractive color variation, including colorless, green, yellow and purple varieties. Often, these colors are graded across a single specimen, as different impurities responsible for the color are consumed from the environment as that specimen crystalizes. Here, we examined samples of both natural green and natural purple fluorite. The purple fluorite specimen was found to not adsorb RNA. In contrast, the green fluorite specimen adsorbed about 25% of the RNA. The green color is often attributed to small amounts of iron or samarium within the mineral lattice. This difference, although observed on only two different specimens from two different sources, is cautionary.

## Materials and Methods

### Radiolabeled RNA

For this study, a 83-nt long labeled RNA molecule was produced by in vitro transcription and isolated by gel electrophoresis. Its sequence was:

5’-^32^P-CGCUGUACGCAACACAAGGCUUAUGGUGUAUCCUCCUGGAUCACGUGUGGUACGUACUGUCCGAUUAUUUCUAAUCGGGAUAC-3’. Data suggest that this RNA may fold into a rod-like, stem-loop structure including three bulges separated by four stems (data not shown, Biondi et al., in preparation).

### Preparation of samples of synthetic minerals

Double decomposition reactions to obtain synthetic minerals by precipitation were prepared by mixing 100 μL each of 100 mM solutions; these gave the synthetic minerals used to collect the data given in Table 1. After precipitation (precipitation times varied from 5 min to a few days), pellets were produced by brief centrifugation, the supernatant was discarded, and 50 μL RNA ≈0.1–0.15 nM were added. RNA was incubated with the mineral for 5 min, after which samples were briefly centrifuged and the supernatant collected for scintillation counter reading. Each pellet was then washed twice with 500 μL H_2_O, once briefly, and once overnight. All fractions were read at the scintillation counter by Cherenkov counting. Percent adsorption was calculated dividing the amount of radioactivity remained in the pellet by the sum of radioactivity in all the washes, multiplied by 100.

In another set of experiments, pellets were lyophilized and weighed prior to the addition of labeled RNA (100 fmol/mg of precipitated mineral). Unfortunately, this approach was not successful for comparative purposes, due to two opposite effects. In some cases (especially with carbonates), the facility with which precipitate minerals redissolved in aqueous RNA solutions prevented any possibility for measurements. In other cases, the increased generic adsorption of aqueous solutions by dry surfaces allowed the powdered minerals to retain all the RNA added regardless of the interactions specific to the mineral (data not shown).

We also collected data for adsorption on precipitated minerals formed at different starting pHs, with values taken before mixing the salts, after the precipitate is formed (pH of the supernatant), and after the RNA is adsorbed (pH of the supernatant) (data not shown). The observations did not alter the conclusions of this paper and were thus omitted.

### Adsorption of RNA on natural minerals

All natural minerals used where from the Benner collection (Table 3). Prior to RNA adsorption, minerals were washed with (in this order) tap water, ddH_2_O, 30% H_2_O_2_, ddH_2_O, EtOH 99%. Minerals were then air-dried for about 30 min in a sterile environment.

**Table 3:** Listed are the origins of each mineral, in alphabetical order.

Mineral	Origin

adamite	Ojuela Mine, Mapimi, Durango, Mexico
agate	location unknown
amazonite	Crystal Peak district, Teller County, CO, USA
apatite	Liscombe Deposit, Wilberforce, Ontario, Canada
aragonite	Atlas Mountains, Morocco
baryte	Sulcis, Sardinia, Italy
beryl	Hunza Mine, Gilit, Pakistan
calcite	a specimen of "Iceland spar", Iceland
celestine	N’Chwaning Mine, Kuruman, South Africa
colemanite	Death Valley, Inyo County, CA, USA
danburite	San Sebastian Mine, Charcas, Mun. de Charcas, San Luis Potosí, Mexico
erythrite	unknown mine, Morocco
green fluorite	Cave in Rock, Hardin county, IL, USA
gypsum	Naica Mine, Chihuahua, Mexico
hematite	Mesabi Range, MN, USA
herkimer diamond	Quartz, Herkimer, NY, USA
magnesite	Minas Gerias, Brazil
magnetite	location unknown
mica	North Carolina, USA
obsidian	location unknown
olivine	(peridotite) Pakistan
opal	Queensland, Australia
ourple fluorite	Cave in Rock, Hardin county, IL, USA
pyrite	Madoc, Ontario, Canada
rhodochrosite	Perú
rutile	Minas Gerias, Brazil
smithsonite	Kelly Mine, NM, USA
strontianite	Winfield Quarry, Winfield Union County, PA, USA
talc	Canada Talc Mine, Madoc, Ontario Canada
topaz	Minas Gerias, Brazil
tourmaline	Minas Gerias, Brazil
vanadinite	Taouz, Er Rachida Province, Morocco
vivianite	Tomokoni mine, Machacamarca District, Potosí, Bolivia
witherite	Cave in Rock, Hardin county, IL, USA

For each mineral, droplets (2 μL) containing ≈100 fmol of radiolabeled RNA were spotted on the surface and let adsorb for 45 min at room temperature. Macro-surface areas of the droplets were obtained with the program ImageJ (NIH). Subsequently, H_2_O droplets of increasing sizes (10 μL to 100 μL) were used to wash the area were the RNA was spotted, until no radioactivity could be detected in the washes. All fractions were then read at the scintillation counter, along with 2 μL of the radioactive RNA originally used. The amount of RNA adsorbed was calculated by subtracting the cpm in all the washes from the cpm of the original 2 μL. In the case of vanadinite, the mineral piece was small enough to fit directly into a scintillation vial, allowing the direct measurement of the radioactivity bound to the piece. This compared to the subtration method showed that the latter was accurate to within ±5%.

### Competitive adsorption of RNA on two competing minerals

To obtain carbonate columns, either of two methods was used. In the first, each carbonate was prepared separately by mixing 1 mL of a 1 M aqueous solution of the chloride (x = Mg/Ca/Sr/Ba) with 1 mL of 1 M Na_2_CO_3_. Two of the carbonates were then combined into a 5 mL chromatographic column. In the second, the chloride salts of two competing metal species were mixed first (1 mL 1 M each) and then let react with 2 mL of 1 M Na_2_CO_3_; in this method, the two minerals co-precipitated, allowing the formation of ternary carbonates that contained two metals together (for example, dolomite is a well-known magnesium calcium carbonate).

With either method, after formation of a precipitate, ≈1 pmol of 5’-^32^P-labeled RNA in 1 mL of H_2_O was added to the mixture. The RNA was allowed to interact with the minerals by 40 min tumbling at room temperature (rt). After this time, each column was set upright in a undisturbed environment for about 15–20 hours to allow the different minerals with different densities to separate.

Autoradiography of the RNA in the columns was obtained by setting a phosphorimager screen (BioRad) tightly against the row of columns in their rack, with the aid of paper clips and weights, for 2 hours in a dark room. Screens were scanned with a Personal Molecular Imager (PMI) phosphorimager (BioRad) and analyzed with the software QuantityOne (BioRad).

After removing the supernatant, carbonate columns were then quickly frozen in liquid nitrogen, extruded from the plastic container by tapping, set against a sterile ruler, and quickly sliced into 0.5–1 cm slices. These were finally passed into clean tubes and radioactive counts in each slide were read at the scintillation counter with the Cherenkov method.

### Feigl’s staining and X-ray powder diffraction

Feigl’s stain [29] is a solution of silver and manganese sulfates. The stain colors orthorhombic and hexagonal carbonates black, but does not stain trigonal carbonates, in the first 30–60 minutes. The reagent was made with 1% silver sulfate (w/v) and 12% manganese sulfate in H_2_O. In the staining experiments, samples were obtained by double decomposition reaction by mixing aqueous solutions of Na_2_CO_3_ (200 µL, 1 M) and CaCl_2_ (200 µL, 1 M). Samples were pelleted, supernatants discharged, and Feigl’s stain (400 µL) was added with vortexing.

The mixtures were then incubated at room temperature, where development of gray color was monitored for up to 3 days. Samples that turned gray generally started developing color after about 20 minutes, while samples that were unstained (white) remained such for the duration of the monitoring period.

### Temperature stability of RNA adsorbed onto aragonite surfaces

For these experiments, five small clusters of aragonite were obtained from an original crystal cluster with the use of a hammer. These were extensively washed with tap water, ddH_2_O, 30% H_2_O_2_, ddH_2_O, and then EtOH (99%) to remove all organic species. The specimens were then air-dried under cover for about 30 min.

Droplets (2 μL) containing ≈100 fmol of radiolabeled RNA were spotted on the surface of each crystal. The material was allowed to adsorb with liquid evaporation and by incubating the mineral pieces at 25, 37, 55, 75, or 95 **°**C in a sterile environment for 2 hours. In parallel, the same amounts of RNA were incubated at the same temperatures in 1.5 mL low-binding test tubes.

After incubation, RNA adsorbed to aragonite surfaces, or adhering to the tubes’ plastic, was released by washing the surfaces with 100 mM aqueous formic acid (100 µL); the released RNA was recovered in new tubes. These samples were subjected to three cycles of evaporation and resuspension in ddH_2_O to eliminate formic acid. The residue was then dissolved in 95% formamide gel loading buffer for denaturing PAGE analysis (18%, 7 M urea). Gels were dried for 30 min at 80 °C before being exposed to a phosphorimager screen for quantitative autoradiography.

### Mineral identification with X-ray powder diffraction

Identification of synthetic minerals was conducted with a power X-ray diffractometer equipped with a copper target (X-Pert Powder; Philips Co.). All diffraction profiles were obtained at a step size of 0.01°, with a divergence and receiving slit of 1° and 0.3 mm, respectively.
